# Covid-19 Barrier gestures for patients with schizophrenia: A therapeutic “dead end”?

**DOI:** 10.1192/j.eurpsy.2022.1289

**Published:** 2022-09-01

**Authors:** I. Bouguerra, E. Khelifa, B. Abassi, S. Ben Aissa, O. Maatouk, N. Kouki, L. Mnif

**Affiliations:** 1 Razi Hospital, F Adult Psychiatry Department, Manouba, Tunisia; 2 Razi, Skolly, Manouba, Tunisia; 3 Hôpital Razi, Psychiatry F, Manouba, Tunisia

**Keywords:** Covid-19, masks, schizophrénia, washing hands

## Abstract

**Introduction:**

Since the beginning of Coronavirus pandemic, the world is facing huge challenges for the prevention of mass infection. Studies shows that wearing facemasks and hand washing seems to be the best protection from disease transmission. Indeed, the spread of SARS-CoV-2 was efficaciously controlled in countries where mask wearing is respected. However, such recommendations may not be easily established with inpatients with mental disorders due to limited ability to embrace instructions.

**Objectives:**

The purpose of this study was to evaluate the use of facemasks and hand’s wash among inpatients with mental disorders during coronavirus pandemic in a psychiatric hospital in Tunisia.

**Methods:**

This hospital-based cross-sectional study was conducted from September to October 2021 among thirty hospitalized inpatients in a psychiatric department suffering from schizophrenia. All patients responded to an anonymous questionnaire about mask wearing and washing hands status. Knowledge about COVID-19 was assessed by a 6-item questionnaire inspired from a Corean study.

**Results:**

Preliminary findings showed that most patients are aware of covid-19 pandemic and about barrier gestures but only a very few (less than 20%) wear masks. Inpatients with schizophrenia were in most cases not afraid from covid-19 contamination within the hospital and less that 50% were vaccinated.

**Conclusions:**

During a pandemic, psychiatric departments needs an extra attention because of the high-risk of spreading infections in inpatients with mental diseases. Communication has to be clearer, and repeated assistance by correctly reminding measures has to be implanted above all for patients with schizophrenia.

**Disclosure:**

No significant relationships.

